# Annotations

**Published:** 1907-07-20

**Authors:** 


### Annotations.

### The Late Sir William Broadbent, Bart.

The main facts of the late Sir William Broadbent's great career are well within the knowledge of our readers, and his name and achievements are certain of their due position in professional history. It may here be permissible to offer one or two reflections on those aspects of his life and work which can only be fully appreciated by those of his own day and generation. There can be nothing but admiration for the steady and resolute course of work which gradually secured for him the confidence of his brethren and general recognition as a great physician. Throughout his career he devoted himself to the duties and obligations of his profession with sincere and single-hearted earnestness, and it is noteworthy that out of this, and out of this alone, there came a great and honourable name. The rewards offered by medicine may not be so abundant as those possible in other professions, but they are at least open to capacity and courage, and it is satisfactory to recognise that qualities such as Sir William Broadbent exhibited, even when unassociated with extraneous assistance, may command appropriate recognition. It is a welcome thought that in the story of his life may be read the possibilities of resolute and unwavering devotion to the opportunities and duties of professional life. This consideration is the more pleasing seeing that he regarded his sphere of action in no narrow or restricted spirit. It is not as one given over to a limited specialism that his name will be remembered. Rather he figures as a conspicuous illustration of the broad and healthy mind to whom all the problems of medicine are both open and interdependent. And this, without question, is one of the secret explanations of the large range and sphere of his influence. With this liberal and informed outlook were associated a robust and sterling common sense and a keen appreciation both of the practical demands and sympathetic relationship of medical work. It was such a union of qualities that made him a great physician. And, in farewell, we cannot but recall that amidst all his honours and duties he found time and interest for the position of his less fortunate brethren. Some of this is written in public record, but much has no chronicle save in grateful hearts and tender memories.

### An Educational Health and Food Campaign.

These are the days of organised efforts and co-operative schemes. A platform, a policy, and an imposing list of patrons, form the usual machinery by which public attention and public support are attracted. It is, one may almost say, a necessity of the time. There can, therefore, be no surprise, and still less complaint, at the application of this method to the modification or reform of our food habits. Some will even recognise an appreciation of the fitness of things in the selection of the Mansion House as the place at which the new campaign makes its first public proclamation. In any event we quite agree that the recently instituted Food Reform League is deserving of much sympathy, and provided it is not annexed by the faddists, we believe it may accomplish a great deal of good. Personally, we have never been able to recognise the claim of the physiologists to regulate the food habits of their fellow creatures, as food, at least so it seems to us, has functions far beyond those that can be estimated in the laboratory. But, without question, there can be nothing but advantage in spreading a knowledge of the economic values of various food stuffs, and in cultivating the art of cookery among the less-informed members of the community. The League does not pretend to advocate any special system of diet, but it proposes to diffuse information regarding the economic and nutritive value of certain foods, which do not, for various reasons, enjoy public favour. The educational efforts of the League will, it is anticipated, be productive of a general tendency to rational feeding and to the cultivation of the "simple and strenuous life."

### The Risks of Factory Workers.

The annual report of the Chief Inspector of Factories and Workshops for 1906 contains a number of interesting, though somewhat anxious, statements. The number of persons employed in factories coming under official review was, approximately, 5,000,000, and among these the accidents reported during the year were 111,904, an increase of 11.2 per cent. over the figures for the preceding year. In fatal accidents the increase was 5.0 per cent. Among various official inquiries allusion is made to five formal investigations conducted by the Board of Trade in connection with boiler explosions; three of these explosions were found to be due to the neglect of the owner, and two to the fault of employees. In the report on industrial poisoning, an increase both of the number of cases and of those having a fatal issue is reported. In 1905 the instances of lead poisoning were 592 with 23 deaths, while last year the figures were 632 with 33 deaths. The occupations in which these cases of lead poisoning occurred were workers in china and earthenware 107 cases, coach-painting 85, whitelead workers 108, file cutting 15, and smelting 38. As compared with this statement it is pleasant to note that during the year only a single case of phosphorus poisoning was reported. On the other hand there were no fewer than 67 cases of anthrax, and of these 22 proved fatal; in 1905 the figures under this section were 59 cases, with 18 deaths. All this provides a very painful illustration of the real risks run by the industrial classes when engaged in the pursuit of their livelihood. Another impressive feature of the report is the increase it records in the number of children and young persons presented for employment in factories. The number examined for this purpose was 390,869, an increase on the previous year of 29,291; of these 42,613 were half-timers, an increase of 1,453. Sanitary defects are, as was to be expected, principally to be found in small premises, many of which are structurally unsuitable for the work carried on. The Department is actively concerned with the ventilation of factories generally, and promises a report on this subject in the course of the year.

